# FHIR Genomics: enabling standardization for precision medicine use cases

**DOI:** 10.1038/s41525-020-0115-6

**Published:** 2020-03-18

**Authors:** Gil Alterovitz, Bret Heale, James Jones, David Kreda, Fan Lin, Lei Liu, Xin Liu, Kenneth D. Mandl, David W. Poloway, Rachel Ramoni, Alex Wagner, Jeremy L. Warner

**Affiliations:** 1 Computational Health Informatics Program, Boston Children’s Hospital, Boston, MA USA; 2 Harvard/MIT Division of Health Sciences and Technology, Harvard Medical School, Boston, MA USA; 3 Intermountain Healthcare, Salt Lake City, UT USA; 4 Center for Biomedical Informatics, Harvard Medical School, Boston, MA USA; 5 Xiamen University, School of Software, Xiamen, Fujian China; 6 Departments of Pediatrics and Biomedical Informatics, Harvard Medical School, Boston, MA USA; 7 Partners HealthCare, Boston, MA USA; 8 Division of Oncology, Washington University School of Medicine, St. Louis, MO USA; 9 Departments of Biomedical Informatics and Medicine, Vanderbilt University, Nashville, TN USA

**Keywords:** Clinical genetics, High-throughput screening, Data processing, Genomics, Health policy

## Abstract

The development of Fast Healthcare Interoperability Resources (FHIR) Genomics, a feasible and efficient method for exchanging complex clinical genomic data and interpretations, is described. FHIR Genomics is a subset of the emerging Health Level 7 FHIR standard and targets data from increasingly available technologies such as next-generation sequencing. Much care and integration of feedback have been taken to ease implementation, facilitate wide-scale interoperability, and enable modern app development toward a complete precision medicine standard. A new use case, the integration of the Variant Interpretation for Cancer Consortium (VICC) “meta-knowledgebase” into a third-party application, is described.

## Introduction

Successful practice of precision medicine will depend upon knowledge-based interpretation of genomic variant data at the point of care, leading to drastically improved diagnosis, prognosis, and treatment selection.^1–4^ Variant data are identified both by traditional genetic panels and high-throughput sequencing, with many large genomic databases already housing non-compatible data structures, often employing codes based on different nomenclatures.^5–7^ It follows that an effective standard is needed to assimilate genomic results from various formats with other clinical data. Currently, most genetic test reports entered into the Electronic Health Record (EHR) are in PDF format.^8^ There is a great opportunity to expand functionality with the recently developed SMART platform utilizing the Fast Healthcare Interoperability Resources (FHIR) specification, enabling apps to be launched directly from within the EHR.

FHIR consists of multiple linkable and extendable data structure specifications called resources, modeling concepts in healthcare scenarios such as patients, conditions, and clinical observations and reports. These resources can be tailored to use cases by standardized sets of constraints and extensions called profiles. This approach has been praised both by large EHR vendors and major technology companies.^9–12^ Given this critical mass and building on the work of Alterovitz et al.^13^, the authors and others in the Health Level Seven (HL7) community have enumerated many clinical genomics use cases in a domain analysis model, including clinical sequencing, cancer screening, pharmacogenomics, public health reporting, and decision support tools.^14^ These use cases and others required the introduction of additional specific data structures to expand the FHIR data model and prototype apps to showcase interoperability.

## Results

FIHR Genomics introduces

1. A new resource, MolecularSequence, for capturing non-interpretive (raw) sequencing data or pointing to it stored in an external repository as needed.
2. New profiles on the existing resources Observation, DiagnosticReport, ServiceRequest, Task, and FamilyMemberHistory to facilitate sharing genetic test results, including observed variants from reference sequences and their clinical implications and interpretations.

A full description of the FHIR Genomics artifacts and suggested usage can be found at https://www.hl7.org/fhir/genomics.html.

The genomics artifacts have been subject to ballot and are deemed ready for trial use implementation. International pilot evaluators from across the spectrum of stakeholders—including hospitals, universities, and vendors—have experimented with FHIR-based clinical genomics apps and use cases.^13,15^ Regular development and testing opportunities arise during international FHIR “Connectathon” events, held three times a year. These events create short feedback loops where research institutions, production EHR systems, and other developers join the community and test the specification for necessary interoperability and features. Further trial use and feedback will result in increasing maturity of the elements within FHIR as they move toward normative status, where adoption can occur without future iterative developments causing breaking changes.

A FHIR Genomics implementation guide has also received HL7 ballot feedback, leveraging standardized “components” within Observation, containing coordinated “codes” and “values.” Codifying individual concepts with this feature facilitates search operations and eases implementation. The guide leverages more profiles on top of the resources versioned in FHIR Release 4, introducing a hierarchical structure of profiles and examples for many use cases.

## Discussion

**Fig. 1:**
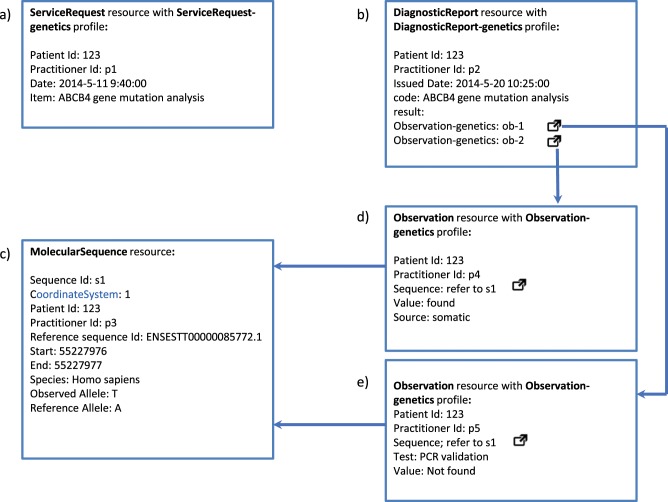
FHIR Genomics workflow use case. **a** A *ServiceRequest* instance; **b** a *DiagnosticReport* instance refers to *Observations* under “result”; **c** a *MolecularSequence* instance carries the reference sequence and variant information; **d** an *Observation* instance carries clinical interpretation; **e** additional *Observations* can carry further analysis information. Arrows depict the inter-resource pointers.

During the domain analysis, it was observed that sequencing data may often need reanalysis, either when delegated to different organizations or determined necessary after updated interpretive information (e.g., on drugs and diseases) becomes available. Using FHIR for genomics data provides corresponding reusability; new resource instances can easily be linked to existing ones thanks to FHIR’s JSON/XML-based architecture and RESTful application program interface (API). This allows for simple implementation, small payload sizes, and intuitive non-duplicative retrieval of data. Figure 1 shows an example depicting a common workflow of FHIR Genomics from an EHR perspective—an order is requested, and test results are reported.

**Fig. 2:**
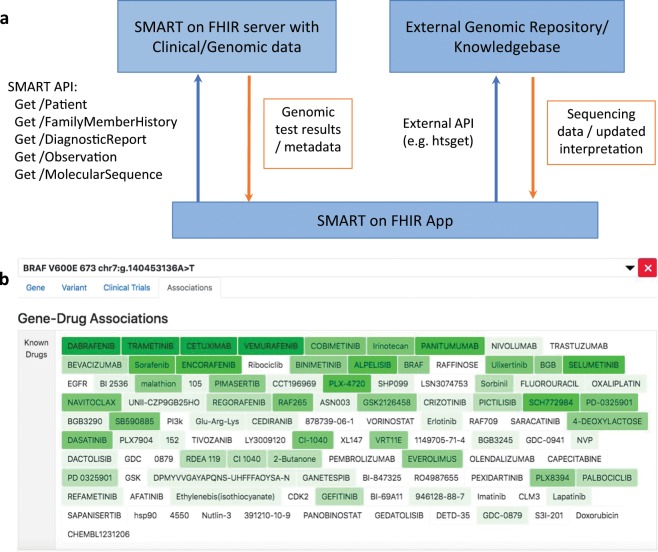
Example of SMART on FHIR application. **a** Arrows show API calls integrating clinical and genomic data at the point of care with external information, for example, through the GA4GH streaming standard htsget.^33^ **b** Screenshot of sample application showing associated drugs in the VICC meta-KB^22^ for a given variant on a sample patient. Clicking a drug name provides a list of relevant publications, sorted by AMP/ASCO/CAP guideline levels of evidence.^23^

Mirroring the common approach of a Picture Archiving and Communication System, it is proposed that EHRs will store only metadata—such as path, address, and IDs—and then retrieve raw sequencing data as needed, forming a Genomic Archiving Communication System (GACS). This approach takes into consideration the large size and limited clinical application of raw genomic data, as well as the trend of new infrastructure being released to the research community before clinical optimization.^16^ Following this model, *MolecularSequence* enables GACS integration with its repository feature, which can carry URIs and identifiers needed to retrieve sequencing data stored in specifications maintained by the Global Alliance for Genomics and Health (GA4GH), for example. A similar approach may also be taken with genomic knowledge-based artifacts as scientific evidence constantly increases. Following ref. ^15^, an FHIR Genomics app may take a patient’s clinical context and observed variations and link this information to external databases to ease interpretation (see Fig. 2).

Compared to other clinical data standards able to communicate genomic information, FHIR Genomics stands out for its inter-resource linkage capabilities and separation of interpretive and non-interpretive data. Before FHIR, HL7 v2 was the healthcare information exchange standard and was extended for genetics test reports with an implementation guide.^17^ However, criticism was made regarding the “Segment” structure—segments were not uniformly created and could lack unique identities, resulting in inefficiencies for data mining, downstream analysis, and complex interoperability. Concerns were also held against the rigid HL7 v3 standard,^18^ where one must largely implement the entire model to transmit data.

Apps can easily retrieve data using services such as SAMtools^19^ and export them into FHIR’s normative and widely adopted Observation resource using the defined genomic profiles. For communication with EHRs, clinicians, patients, and decision support engines, scaling this conversion (e.g., to all variants detected from whole-genome sequencing) may stress systems, so filtering for clinical actionability and/or translating on demand from a GACS implementation may be needed. Servers storing FHIR Genomics resources can interface with the research community through GA4GH APIs such as Beacon,^20^ though careful data security considerations must be made, as these servers may also contain additional sensitive clinical information.

FHIR Genomics has been shown to represent genomic data that suits the needs of current and upcoming clinical genomics use cases, and SMART technology continues to translate the potential of a single data standard into powerful precision medicine apps. As envisioned, the FHIR Genomics framework enables many value-added opportunities such as clinically integrated genomics knowledge-based apps and a translational bridge between research-oriented genomics data and precision medicine.

## Methods

To further probe the capabilities of the FHIR Genomics guidance, a prototype app was expanded to read and record genomic variants per updates to the FHIR R4 standard and implementation guide.^21^ In addition, cancer variants were linked to the Variant Interpretation for Cancer Consortium meta-knowledgebase API^22^ for context, with evidence classified by the AMP/ASCO/CAP somatic classification guidelines.^23^ A reference server was constructed using the Health Services Platform Consortium sandbox (https://sandbox.hspconsortium.org) where the app was successfully integrated with sample patient data. Additional supported use cases require storage and validation of information and terminologies cataloged by diverse efforts in the genomics research community, including ClinVar identifiers,^24^ HGVS nomenclature,^25^ HUGO Gene Nomenclature Committee identifiers,^26^ NCBI reference sequences,^27^ terms from the Sequence Ontology,^28^ clinical guidelines,^29,30^ and services, such as LOINC^31^ and SNOMED CT.^32^

### Reporting summary

Further information on research design is available in the Nature Research Reporting Summary linked to this article.

## Supplementary information

Reporting Summary

## Data Availability

All relevant data are available from the authors without restriction. Guidance for the data structures needed for FHIR genomics can be found at http://hl7.org/fhir/R4/genomics.html and http://hl7.org/fhir/uv/genomics-reporting/index.html.

## Code availability

Code for the updated reference SMART-on-FHIR application is freely available on GitHub via https://github.com/smart-cancer-navigator/Application, with a public instance and instructions for linking to the public HSPC sandbox hosted at https://smart-cancer-navigator.github.io/app. Python code for a prototype reference server with sample FHIR genomics data is also available on GitHub at https://github.com/bcl-lab/FHIR-Genomics_v2.
